# Spatio-temporal evolution of sports tourism industry integration: A empirical analysis of Xinjiang in China

**DOI:** 10.1371/journal.pone.0300959

**Published:** 2024-04-10

**Authors:** Ke Zhang, Xuehui Mei, Zhengqing Xiao

**Affiliations:** College of Mathematics and System Science, Xinjiang University, Urumqi, China; Rikkyo University, JAPAN

## Abstract

Since the issuance of the "Guiding Opinions on Vigorously Developing Sports Tourism" in 2016, the integration of sports and tourism has become a strategy in regional economic development. It creates new economic growth points, enhances local images, and promotes cultural communication. In the context of the "Tourism Makes Xinjiang Thrive" strategy, quantitatively investigating the integration of the sports and tourism industries helps people to better understand their interaction which can serve as the valuable input in policy-making for the comprehensive development of a region. This paper uses entropy weight method, stochastic frontier analysis and coupling coordination model to quantitatively analyze the effect of sports tourism industry integration in Xinjiang from the perspective of integration path. Meanwhile, the Dagum Gini coefficient and nuclear density estimation were used to analyze the regional differences and dynamic evolution of industrial integration quality. The result shows that (1) The sports and tourism integration quality in Xinjiang has not reached the optimal goal of complete integration. In the process of mutual industrial promotion, tourism promotes a higher degree of integration with the sports industry. (2) The industrial integration quality shows a phenomenon of “imbalance and inadequacy” among the regions. The regions with high quality of industrial integration were Urumqi, Ili, Kashgar, Altay and Changji, which have rich sports tourism resources. (3) The overall spatial difference in the quality of industrial integration presented a fluctuation downtrend. The difference between the tourism industrial belts was very significant.

## Introduction

In recent years, people’s demand for diversified sports and tourism leisure has been growing day by day. Under the background of global tourism and national fitness, the sports tourism integration ushered in a new opportunity across the board from top-level design, overall layout to market-driven development [1]. As a province with the largest terrain and rich tourism resources in China, and unique natural scenery and traditional culture, Xinjiang has laid a solid foundation for the development of sports tourism industry [2].

Since the successful hosting of the Beijing Olympic Games in 2008, the sports tourism industrial integration has become the mainstream research for experts in this field [3]. By summarizing the studies of relevant scholars, it is found that the current research perspectives mostly focus on the path of industrial integration [4, 5], integration mode [6, 7], integration power [8, 9], era value [10, 11] and so on. For example, Li and Luo (2019) constructed the integrative development path from industrial chain, including policy coordination, resource pooling and market docking [12]. According to the purpose and way of participation, Mokras-Grabowska (2016) categorized the integration mode of sports tourism industry into four forms: fan tourism, nostalgia, sport event participation and leisure [13]. Yang (2016) believed that the dynamic mechanism of industrial integration comes from the asset versatility of sports resources and the driving force of tourism consumption structure [14]. Perić et al. proposed the business model applicable to outdoor activity tourists and recommended that the professionals develop appropriate business strategies to better meet the needs of sports event tourism [15]. Khan (2018) took Cambridge City as an example to analyze the role and influence of urban sports tourism development in social, economic, cultural, environmental and other aspects, and concluded that the importance of sports tourism is constantly improving [16]. Weed and Streimikiene et al. analyzed the challenges faced by the tourism industry during the COVID-19 pandemic and the role played by the integration of sports and tourism [17, 18]. In addition, some other scholars have studied the industrial integration at the regional level, based on the comparative advantages of regional characteristics and resource structure. Malchrowicz-Mosko and Poczta (2018) thought that the organization of small sports events and events related to cultural heritage in specific areas is conducive to improving local image [19]. Ge and Li (2022) analyzed the influencing factors of ice and snow tourism brand construction in Jilin Province, and put forward constructive strategies [20].

Based on the gradual improvement of the industrial integration theory, some experts and scholars began to investigate the measurement of the integration level of sports and tourism industries. Xu and Chen (2020) constructed an evaluation index system of industrial integration of sports and tourism, and discussed its temporal and spatial characteristics [21]. Li et al. (2021) applied the coupling coordination model and the GeoDetector to calculate integration level of sports tourism and the factors affecting the coordinated development [22]. Yang et al. (2023) used the benevolent cross-efficiency DEA model to calculate sports tourism integration efficiency, and conducted influencing factors analysis by the qualitative comparative analysis [23]. Furthermore, Yang et al. proposed a new hybrid MCDM model that can be transformed into a scientific quantitative analysis based on qualitative investigations conducted by experts [24, 25]. Their research has successfully explored four dimensions of sustainable sports tourism development.

The above researches provided theoretical basis and reference model for the integrative development. The integration of sports tourism has also achieved certain results. However, the industrial integration faces such problems as insufficiency of integrative depth, lack of industrial support ability, and weak driving force of industrial development [26]. Therefore, people started to wonder how to measure integration quality of sports and tourism industries. Fu and Yang (2020) thought that focusing on technical efficiency could achieve high-quality tourism development [27]. Xie et al. (2012) applied the stochastic frontier analysis in studying the integration of industrialization and informatization for the first time, and discussed the quality of integration of industrialization and informatization in China [28]. Zhang et al. (2023) measured the integration quality of Chinese cultural tourism industry from the perspective of technical efficiency, and explored its influencing factors and formation mechanism [29]. Based on the above research on the quality of industrial integration, this paper defines the concept of industrial integration quality, which is the process of the interaction and promotion between sports and tourism industries to achieve technical efficiency.

The tourism is one of the industries with obvious industrial clustering, so the industrial belt of spatial connection is an inevitable choice for the tourism industry to adapt to economic development. Shen et al. (2016) analyzed the status of sports culture tourism industrial belt in Beijing and Zhangjiakou, and proposed strategies to accelerate the development of the tourism industrial belt [30]. Yang et al. (2017) used the fractal diffusion limited aggregation (DLA) principle to conduct the DLA spatial analysis on A-level landscape clusters, and deconstructed the spatial fractal mechanism of the synergistic development of Eco-tourism industrial belt and urban clusters [31]. The formation of the tourism industrial belt depends on the polarization and diffusion of urban agglomeration, and its goal is to make the tourism industry elements exert their agglomeration and fan-out effect, which has great practical significance for optimizing regional tourism industry structure and stimulating steady economic growth [32]. Therefore, people can develop diversified sports tourism products according to the regional characteristics of the tourism industrial belt, and realize the effective allocation of sports tourism resources.

The Gini coefficient decomposition method based on the Theil index can explain the source of regional differences [33]. Zhang and Zhou (2016) analyzed the gap about the regional innovation level using Dagum Gini coefficient and spatial Markov chain [34]. In addition, kernel density estimation can visually display evolution process of data distribution through three-dimensional evolution graphics. Zhang et al. (2022) used kernel density estimation to describe the dynamic evolution of carbon emission intensity in China’s main strategic regions [35]. To strengthen regional cooperation, we should quantitatively analyze the degree of difference in industrial integration quality in various tourism industrial belts.

To sum up, the existing literatures have reference value for exploring the integrated development of sports and tourism industries, but there are still some limitations:

1. The quantitative analysis of industry integration research is relatively scarce.
2. The integration of sports tourism industry is a two-way dynamic process, and there is no theoretical model to explain the integration path of sports and tourism.
3. Exploring regional differences are mostly based on geographical divisions, lacking regional development analysis from the perspective of tourism industrial belt.
4. Most existing studies are based on static spatio-temporal differences, lacking an analysis on dynamic evolutionary patterns.

In order to make up for these deficiencies, this paper constructed a theoretical model of industrial integration quality. Utilizing entropy weight method, stochastic frontier analysis, and coupled coordination model, a quantitative analysis was conducted to explore the integration quality of the sports tourism industry in 14 regions of Xinjiang from 2010 to 2019. Besides, for the four industrial belts defined in "the 13th Five-year Plan" of the Xinjiang tourism industry, we analyzed their differences and dynamic evolution patterns of sports tourism integration using Dagum Gini coefficient and nuclear density estimation. It is hoped that this study can enrich the research on the integration of sports tourism industry and provide good reference for strengthening regional cooperation and promoting sustainable local economic development.

## Materials and methods

### Methods

#### Theoretical model

The integration of sports and tourism industries is a process in which the two interact and promote each other to achieve technical efficiency. It is manifested that a minimal investment in the tourism industry can bring sports industry benefits, or vice versa. The point of the minimum total investment is industrial integration point of sports tourism.

However, sports tourism integration is often restricted by many factors, such as institutional change, cross-sectoral coordination, investment structure, management costs. These factors constitute the friction cost, which has an impact on the integration process, resulting in the deviation of the actual development level from the ideal deterministic production frontier. In order to reduce the impact of friction costs, industrial integration is often intervened through coordination behaviors such as the government macro-control, technology advances, and market regulations. Therefore, this paper constructs a theoretical model of industrial integration under perfect competition and imperfect competition respectively, which provides theoretical support for the quality evaluation of sports and tourism integration.

1. Industrial integration under perfect competition
  It is assumed that there is no friction cost in industrial integration under perfect competition. To describe the ideal integration state, the integration status of sports tourism at stage n are described as follows:
$$X_{1}^{n}=X_{1}^{n-1}+r_{1}(X_{2}^{n-1}-X_{1}^{n-1})=(1-r_{1})X_{1}^{n-1}+r_{1}X_{2}^{n-1}$$ (1)
$$X_{2}^{n}=X_{2}^{n-1}+r_{2}(X_{1}^{n-1}-X_{2}^{n-1})=(1-r_{2})X_{2}^{n-1}+r_{2}X_{1}^{n-1}$$ (2)
  Where $X_{i}^{j}$ represents the position of tourism (*i* = 1) or sports (*i* = 2) industry in stage j. *r*_1_ is the integration coefficient of tourism system versus sports system, which depicts the integration speed of sports to promote tourism development. *r*_2_ is the integration coefficient of sports system versus tourism system, which depicts the integration speed of tourism to drive sports development.
2. Industrial integration under imperfect competition
  Under the condition of imperfect competition, there are friction costs and coordination costs in industrial integration. The integration status of sports tourism at stage *n* are described as follows:
$$X_{1}^{n}=X_{1}^{n-1}+(r_{1}+\Delta r_{1}+c_{1})(X_{2}^{n-1}-X_{1}^{n-1})$$ (3)
$$X_{2}^{n}=X_{2}^{n-1}+(r_{2}+\Delta r_{2}+c_{2})(X_{1}^{n-1}-X_{2}^{n-1})$$ (4)

Where Δ*r*_1_ and Δ*r*_2_ are the friction cost coefficients existing in sports system and tourism system respectively, both are reasonable negative numbers with absolute values less than 1, and their absolute values are inversely related to the integration level of sports tourism. *c*_1_ and *c*_2_ represent the coordination cost coefficients of intervention in the path deviation of sports system and tourism system respectively, and both are reasonable positive numbers with absolute values are less than Δ*r*_1_ and Δ*r*_2_.

Therefore, assessing sports tourism integration quality could be done by examining the degree of deviation between the actual state and the ideal state in the process of integration in order to reflect the process quality of industrial integration. It can also be examined the impact of industrial integration on macroeconomic indicators to reflect the quality of industrial integration results.

**Fig 1:**
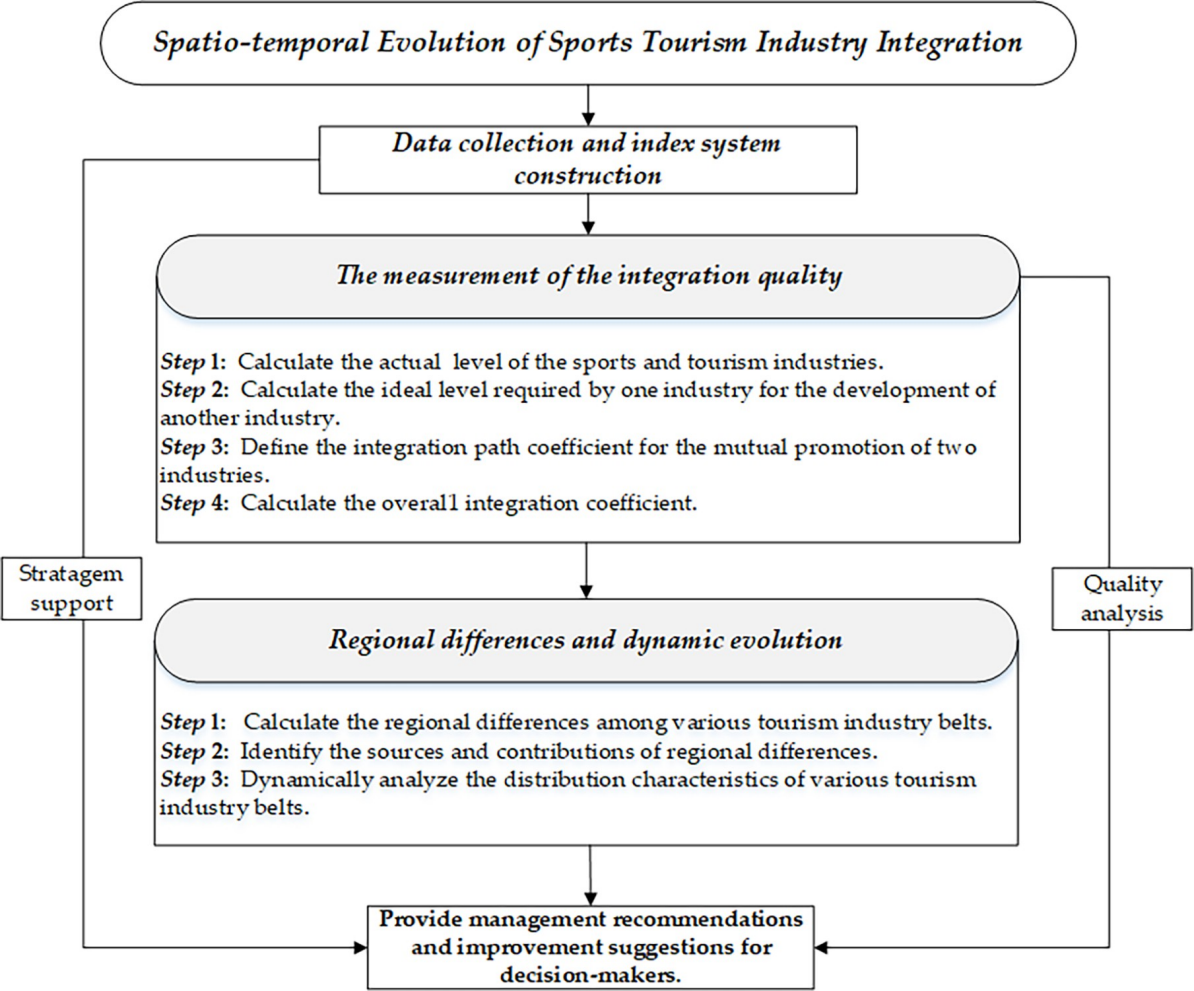
The analysis procedure diagram.

This paper evaluates the integration process from two aspects: one is how sports industry promotes tourism industry and the other is how tourism industry promotes sports industry as two separate paths. Then, it couples these two paths and calculates the level of industrial integration quality in sports tourism, thereby expanding the depth of research in the field of sports tourism integration. Then, further analysis was conducted on the regional variations and dynamic evolution processes of integration quality. Fig 1 illustrates the model and computational steps employed in the study.

#### Entropy method

This paper uses the entropy method to determine the weight of the index and the comprehensive evaluation results, so as to describe the real state of the industrial development. As an objective weighting method, the entropy method can make the evaluation results more reliable and robust. The calculation steps are as follows:

(1) Due to the different measurement units of each index, the original data needs to be dimensionless.
$$X_{ij}^{\prime }=\frac{X_{ij}-min(X_{1j},X_{2j},\ldots ,X_{mj})}{\max\left(X_{1j},X_{2j},\ldots ,X_{mj}\right)-min(X_{1j},X_{2j},\ldots ,X_{mj})}+0.001$$ (5)
  Where *X*_*ij*_ is original value of index *j* in year *i*, *m* represents the number of years, $\max\left(X_{1j},X_{2j},\ldots ,X_{mj}\right)$ and $min(X_{1j},X_{2j},\ldots ,X_{mj})$ are the maximum and minimum values of index *j* in all the years respectively, and $X_{ij}^{\prime }$ represents the standardized data. At the same time, considering that zero value may occur in dimensionless quantization, a small value 0.001 is added.
(2) The entropy value of the index *j*
$$p_{ij}=\frac{X_{ij}^{\prime }}{\sum_{i=1}^{m}X_{ij}^{\prime }}$$ (6)
$$e_{j}=-\frac{1}{ln\;m}\sum_{i=1}^{m}p_{ij}\;ln\;p_{ij}$$ (7)
  Where *p*_*ij*_ represents the proportion of index *j* in year *i*, and *e*_*j*_ represents the entropy.
(3) The weight of the index
$$d_{j}=1-e_{j}$$ (8)
$$w_{j}=\frac{d_{j}}{\sum_{j=1}^{n}d_{j}}$$ (9)
  Where *d*_*j*_ represents the difference coefficient of the index *j*, *n* is the number of indexes, and *w*_*j*_ is the weight of index *j*.
(4) The comprehensive level index is calculated by using the standardized data and the weight.
$$F_{i}=\sum_{j=1}^{n}w_{j}X_{ij}^{\prime }$$ (10)

#### Stochastic frontier analysis model

Stochastic frontier analysis is a method of efficiency estimation using stochastic frontier production function, and it takes into account random errors and uncertainties, making it more flexible and robust in evaluating efficiency. This paper establishes a stochastic frontier analysis model to estimate the ideal state of industrial integration development. We set the model of tourism driving the integration of sports industry as:

$${SPO}_{it}=f({TOU}_{it},i,t)+\epsilon_{it}$$ (11)

Where *SPO*_*it*_ and *TOU*_*it*_ are the actual development level of sports industry and tourism industry of region *i* in year *t* respectively. *f*(*TOU*_*it*_, *i*, *t*) is the ideal development level of sports industry required by the development of tourism industry, which depicts the integration path of the tourism driving the sports, *ϵ*_*it*_ is a random disturbance term.

Similarly, the model of sports promoting the integration of tourism industry is set as:

$${TOU}_{it}=f({SPO}_{it},i,t)+\epsilon_{it}$$ (12)

Through the stochastic frontier analysis method, the Formula (11) and Formula (12) are used for the estimation. On this basis, the integration coefficient of tourism driving sports industry of the region *i* in year *t* is defined as:

$${IC1}_{it}=exp(\hat{f}({TOU}_{it},i,t)-\underset{j=1,..n}{max}\hat{f}({TOU}_{it},j,t))$$ (13)

Here, *n* represents the number of years, the integration of sports industry driven by tourism industry of the region *i* in year *t* reflects the gap between the level of sports industry development required by the development of tourism of the region *i* in year *t* and the maximum possible development level of sports industry required by the same level of tourism industry in all regions at the same time.

Similarly, the integration coefficient of sports promoting tourism of region *i* in year *t* is defined as:

$${IC2}_{it}=exp(\hat{f}({SPO}_{it},i,t)-\underset{j=1,..n}{max}\hat{f}({SPO}_{it},j,t))$$ (14)

#### Coupling coordination model

The coupling coordination degree model can explain the interrelationships between several subsystems, and further comprehensively evaluate the entire system. In this paper, we use this model to calculate the overall integration coefficient. The calculation formula are as follows:

$$C({IC1}_{it},{IC2}_{it})=2\times {\left[\frac{{IC1}_{it}\times {IC2}_{it}}{{\left({IC1}_{it}+{IC2}_{it}\right)}^{2}}\right]}^{\frac{1}{2}}$$ (15)

$$T=\frac{1}{2}{IC1}_{it}+\frac{1}{2}{IC2}_{it}$$ (16)

$$D=\sqrt{C*T}$$ (17)

Where *IC*1_*it*_ and *IC*2_*it*_ respectively represent the integration coefficient of tourism driving sports industry and sports industry promoting tourism of the region *i* in year *t*. *C* is the coupling degree and *T* is the comprehensive evaluation index. Considering the importance of the two integration paths to the coupling relationship, 1/2 weight is assigned to each of the two integration paths. *D* is the coupling coordination degree, and the value range of *D* is 0 to 1. The nearer to 1 the D value approaches, the more well-aligned the two integration paths are developed, and it gets closer to the goal of promoting tourism through sports and driving sports through tourism.

#### Dagum Gini coefficient

The Dagum Gini coefficient can not only effectively identify the source of the gap between regions, but also solve the problem of overlapping between samples. The overall Gini coefficient is calculated as follows:

$$G=\sum_{j=1}^{k}\sum_{h=1}^{k}\sum_{i=1}^{n_{j}}\sum_{r=1}^{n_{h}}|y_{ji}-y_{hr}|/2n^{2}\bar{y}$$ (18)

Where *k* represents the number of regions divided; *y*_*ji*_ and *y*_*hr*_ are the industrial integration quality in prefecture *i* of region *j* and prefecture *r* of region *h* respectively; *n*_*j*_ and *n*_*h*_ are the total number of prefectures in region *j* and region *h*, respectively. *n* represents the total number of prefectures; $\bar{y}$ is the average value of integration quality in all prefectures.

In the decomposing of Gini coefficient, we should sort the average of integration quality of each region, that is, ${\bar{Y}}_{1}\leq \cdots {\bar{Y}}_{j}\leq \cdots {\bar{Y}}_{k}$, where ${\bar{Y}}_{j}$ represents the average of integration quality of prefecture in the region *j*. Then, the overall Gini coefficient can be divided into the following three parts:

1. The Gini coefficient and intra-regional difference in region *j* are as follows:
$$G_{jj}=\frac{1}{2{\bar{Y}}_{j}}\sum_{i=1}^{n_{j}}\sum_{r=1}^{n_{j}}|y_{ji}-y_{jr}|/n_{j}^{2}$$ (19)
$$G_{w}=\sum_{j=1}^{k}G_{jj}p_{j}s_{j}$$ (20)
  Where *p*_*j*_ = *n*_*j*_/*n* indicates the proportion of prefectures in region *j* to the total, and $s_{j}=n_{j}\bar{Y_{j}}/n\bar{Y}$ indicates the proportion of integration quality in region *j* to the total.
2. The Gini coefficient and inter-regional net difference between regions *j* and *h* are as below:
$$G_{jh}=\sum_{i=1}^{n_{j}}\sum_{r=1}^{n_{h}}\left|y_{ji}-y_{hr}|/n_{j}n_{h}({\bar{Y}}_{j}+{\bar{Y}}_{h}\right)$$ (21)
$$G_{nb}=\sum_{j=2}^{k}\sum_{h=1}^{j-1}G_{jh}(p_{j}s_{h}+p_{h}s_{j})D_{jh}$$ (22)
  Where *D*_*jh*_ represents the relative impact of the growth of integration quality between regions *j* and *h*, which can be expressed as follows: $D_{jh}={(d}_{jh}-p_{jh})/{(d}_{jh}+p_{jh})$, *d*_*jh*_ and *p*_*jh*_ are mathematical expected values of the sum of all samples of (*y*_*ji*_−*y*_*hr*_>0) and (*y*_*ji*_−*y*_*hr*_<0) in regions *j* and *h*, respectively.
3. The hypervariable density is:
$$G_{t}=\sum_{j=2}^{k}\sum_{h=1}^{j-1}G_{jh}(p_{j}s_{h}+p_{h}s_{j})(1-D_{jh})$$ (23)

#### Kernel density estimation

The kernel density 3D evolution graphs are more adherent to the actual data space. Through the surfaces and contour lines in the graphs, one can intuitively grasp the clustering and dispersion of data points. In this paper, the 3D evolution graph is used to visually show the changing process of data distribution form, and the overall information is displayed from the number, position, height and curve trailing of peaks.

Specifically, the kernel density curve of industrial integration quality in region *j* is generated by the following function:

$$f_{j}(y)=\frac{1}{n_{j}h}\sum_{i=1}^{n_{j}}K(\frac{y_{ji}-{\bar{y}}_{j}}{h})$$ (24)

Where, *n*_*j*_ is the number of observations, *K*(∙) represents the kernel density function, which describes the weight of all sample points *y*_*ji*_ in the *y* neighborhood. ${\bar{y}}_{j}$ is the average value, and *h* represents the bandwidth. For kernel function selection, we use Gaussian kernel function with higher accuracy as follows:

$$f(x)=\frac{1}{\sqrt{2\pi }}exp(-\frac{x^{2}}{2})$$ (25)

In the process of Kernel density estimation, bandwidth selection is extremely sensitive. Therefore, we need to select the appropriate bandwidth. For the more densely distributed areas, a narrower bandwidth is chosen. In practice, we used cross-validation method to determine the appropriate bandwidth.

### Index system

Based on the principles of scientific validity, applicability, and data accessibility, we constructed an indicator system to analyze the integration quality between sports and tourism industries by referring to the existing relevant literature and taking into account that these indicators should reflect the mechanisms of industry integration and regional differences. The indicators are captured in Table 1.

**Table 1:** Index system of sports tourism industry.

Subsystem	Tier 1 indicators	Tier 2 indicators	Unit	Weight
Sport industry	Production factors	Per capita area of sports venues	Square meter	0.188
		Sports lottery amount	Billion yuan	0.242
		Number of employees in cultural sports and entertainment industry	Person	0.244
		Fixed assets investment by cultural sports and entertainment industry	10,000 yuan	0.236
	Industrial economy	Retail Price Index for Sports products	%	0.013
		The average salary of employees in sports sectors	Yuan	0.077
Tourism industry	Production factors	Total number of travel agencies	Number	0.183
		Number of starred hotels	Number	0.073
		Number of scenic spots above 3A	Number	0.051
		Number of Employees in accommodation catering industry	Person	0.165
		Fixed assets investment in catering and accommodation industry	10,000 yuan	0.172
	Industrial economy	Income of catering and accommodation industry	10,000 yuan	0.235
		Total tourism revenue as a percentage of GDP	%	0.121

According to the availability of data, we draw on the index selection methods of Li et al. (2021) [22], Pang (2023) [36] and Yuan et al. (2022) [37]. We adopts the “number of personnel employed in cultural sports and entertainment industry” and “fixed assets investment by cultural sports and entertainment industry” as the factor index of sports industry. For the explanation of the effectiveness of alternative indicators, Zhang and Wang (2012) pointed out that the sports industry belongs to the culture, sports and entertainment industry, and the cultural connotation and entertainment of the sports industry are strong [38]. Therefore, the relevant indicators of culture, sports and entertainment industry can largely reflect the corresponding indicators of the sports industry.

## Results

### The temporal and spatial evolution characteristics of the integration quality

#### Temporal characteristics analysis

In order to investigate the extent to which sports and tourism play a role in the process of mutual promotion, Eqs (13) and (14) are used to calculate the integration coefficients about how tourism drove sports and sports promoted tourism in different prefectures of Xinjiang from 2010 to 2019, and further calculated the average values of the integration coefficients of the two basic paths respectively (Table 2). In the two basic paths, the integration degree of tourism driving sports industry is significantly higher than that of sports promoting tourism industry.

**Table 2:** Average level of integration coefficient of the two basic paths.

Year	2010	2011	2012	2013	2014	2015	2016	2017	2018	2019
**T→S** ^***a***^	0.517	0.622	0.517	0.667	0.652	0.599	0.603	0.621	0.643	0.751
**S→T** ^***b***^	0.437	0.380	0.363	0.295	0.340	0.428	0.397	0.353	0.366	0.424

a represent integration coefficient of sports promoting tourism, and b represent integration coefficient of tourism driving sports.

On the basis of the integration coefficient of the two basic paths, the overall integration coefficient in the 14 regions of Xinjiang from 2010 to 2019 is estimated by Eqs (15)–(17). The results are captured in Table 3. For most of the regions, the integration quality is generally on the rise.

**Table 3:** Quality level of industry integration in 14 regions.

Year	2010	2011	2012	2013	2014	2015	2016	2017	2018	2019
**Urumqi**	0.891	0.916	0.918	0.928	0.922	0.940	0.925	0.911	0.930	0.931
**Karamay**	0.628	0.619	0.543	0.516	0.625	0.649	0.589	0.559	0.546	0.608
**Turpan**	0.535	0.575	0.502	0.467	0.551	0.636	0.617	0.638	0.624	0.738
**Hami**	0.510	0.566	0.518	0.520	0.554	0.614	0.556	0.526	0.581	0.666
**Changji**	0.574	0.704	0.661	0.611	0.656	0.714	0.720	0.752	0.815	0.878
**Ili**	0.819	0.843	0.857	0.851	0.872	0.890	0.882	0.880	0.892	0.903
**Tacheng**	0.528	0.597	0.559	0.535	0.548	0.584	0.580	0.575	0.541	0.678
**Altay**	0.797	0.850	0.753	0.743	0.787	0.803	0.786	0.777	0.771	0.840
**Bozhou**	0.521	0.561	0.502	0.516	0.546	0.610	0.598	0.517	0.523	0.595
**Bazhou**	0.655	0.748	0.676	0.662	0.746	0.715	0.704	0.721	0.730	0.790
**Aksu**	0.605	0.594	0.599	0.571	0.617	0.616	0.597	0.577	0.606	0.650
**Kezhou**	0.461	0.496	0.459	0.409	0.483	0.513	0.513	0.487	0.508	0.540
**Kashi**	0.745	0.778	0.753	0.673	0.762	0.798	0.795	0.757	0.784	0.822
**Hotan**	0.506	0.554	0.469	0.459	0.513	0.553	0.545	0.538	0.519	0.605
**Average**	0.627	0.672	0.626	0.604	0.656	0.688	0.672	0.658	0.669	0.732

**Fig 2:**
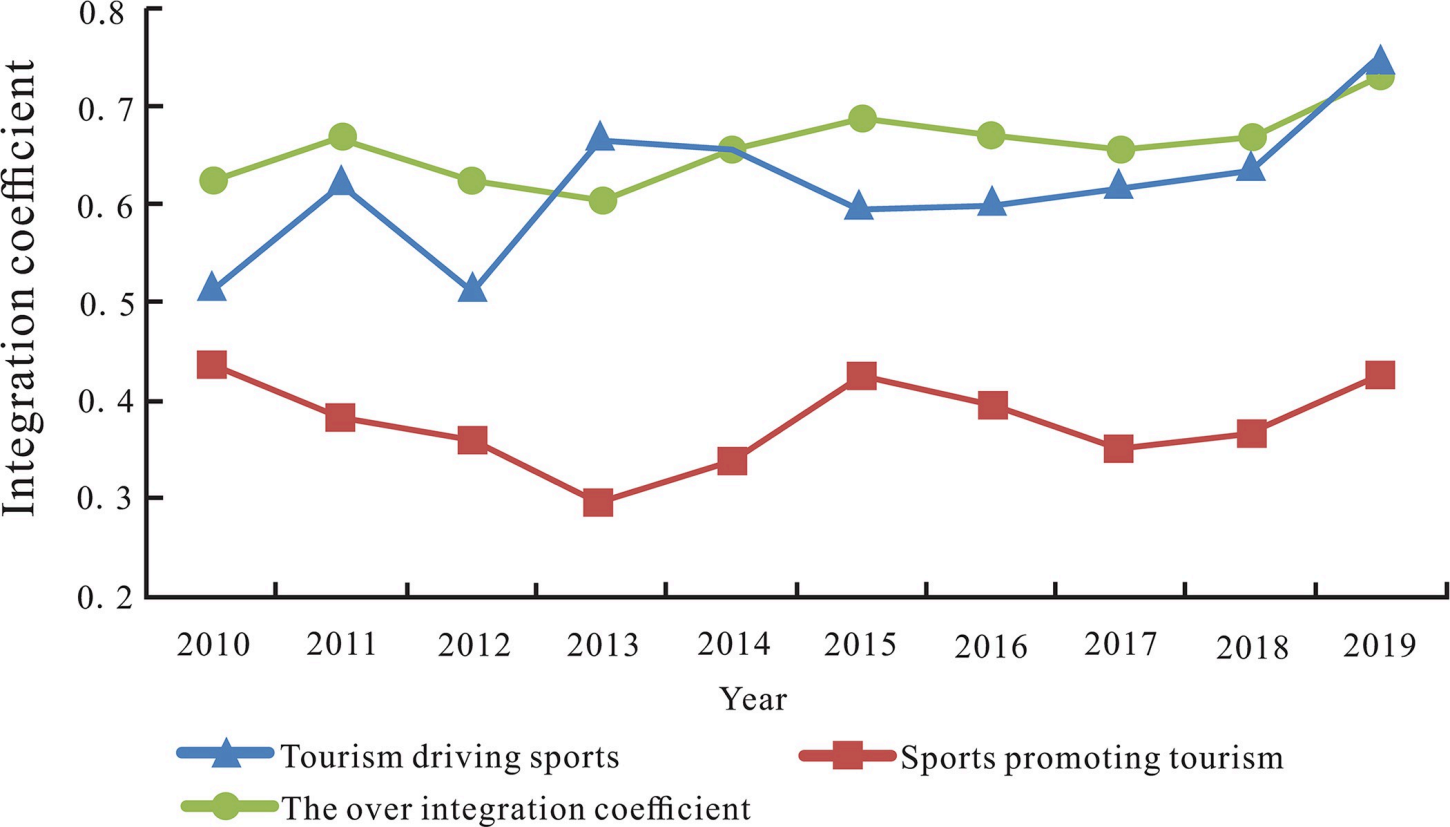
Trend of integration path and integration quality.

Fig 2 is drawn based on Tables 2 and 3 to illustrate the trend. As shown in Fig 2, both the overall industry integration coefficient and the integration coefficient of tourism driving sports show a fluctuating upward trend, while the integration coefficient of the sports promoting tourism was not significantly increased. In addition, the path of tourism driving sports, the path of sports promoting tourism, and the overall industrial integration quality did not reach the optimal level, and there is a certain distance from complete integration (the horizontal line of integration coefficient equal to 1.0).

#### Quality analysis of industrial integration process

According to the theoretical model of industrial integration quality proposed in this paper, there are two sources of the deviation of sports tourism development level: one is the deviation from the stochastic frontier, and the other is the deviation from the ideal level.

1. In order to investigate the deviation of the industrial development level from the stochastic frontier, we define the deviation of the integration of tourism driving sports in Prefecture *i* in year *t* as the gap between the ideal level ($\hat{f}({TOU}_{it},i,t)$) of sports industry required by the tourism development and its corresponding frontier plane ($\underset{j=1,..n}{max}\hat{f}({TOU}_{it},j,t)$). Similarly, the integration deviation of sports promoting the tourism in Prefecture *i* in year *t* is defined as the gap between the ideal level ($\hat{f}({SPO}_{it},i,t)$) of the tourism industry required by the sports development and its corresponding frontier plane ($\underset{j=1,..n}{max}\hat{f}({SPO}_{it},j,t)$). According to Formulas (13) and (14), the deviation of the ideal level from the frontier has the same trend as the integration coefficient of two basic paths. Therefore, the change trend of integration coefficient of the two basic paths in Fig 2 can reflect the change of the deviation extent of the integration path. In general, the path deviation extent of sports promoting tourism is greater than that of tourism driving sports industry integration in the observation period. During the period from 2010 to 2016, the deviation between the two basic paths and the stochastic frontier is not consistent. While the integration paths of tourism driving sports and sports promoting tourism were approaching the stochastic frontier from 2017 to 2019, and the deviation of the two paths was consistent.
2. Then, we investigate the deviation of sports and tourism from their respective ideal level. We define the deviation of sports industry from its ideal level in year *t* of Prefecture *i* as the gap between the actual development level of sports (*SPO*_*it*_) and ideal level of sports ($\hat{f}({TOU}_{it},i,t)$) required by the development of tourism industry. Similarly, the deviation of tourism industry from its ideal level in year *t* of Prefecture *i* is defined as the gap between the actual development level of tourism industry (*TOU*_*it*_) and ideal level of tourism industry ($\hat{f}({SPO}_{it},i,t)$) required by the development of sports industry. By Formula (10), the actual development level of sports industry and tourism industry can be calculated. The ideal level of sports industry required by tourism development and the ideal level of tourism industry required by sports development can be calculated by using Formulas (11) and (12), respectively. Finally, the deviation of sports and tourism industry from their ideal levels are obtained, and the average deviation was further calculated (Table 4).

**Table 4:** Average deviation of sports industry and tourism industry.

Year	2010	2011	2012	2013	2014	2015	2016	2017	2018	2019
Sports deviation	0.052	0.031	0.025	0.020	0.031	0.015	0.011	0.044	0.039	0.020
Tourism deviation	0.019	0.026	0.036	0.063	0.041	0.027	0.025	0.030	0.021	0.016

**Fig 3:**
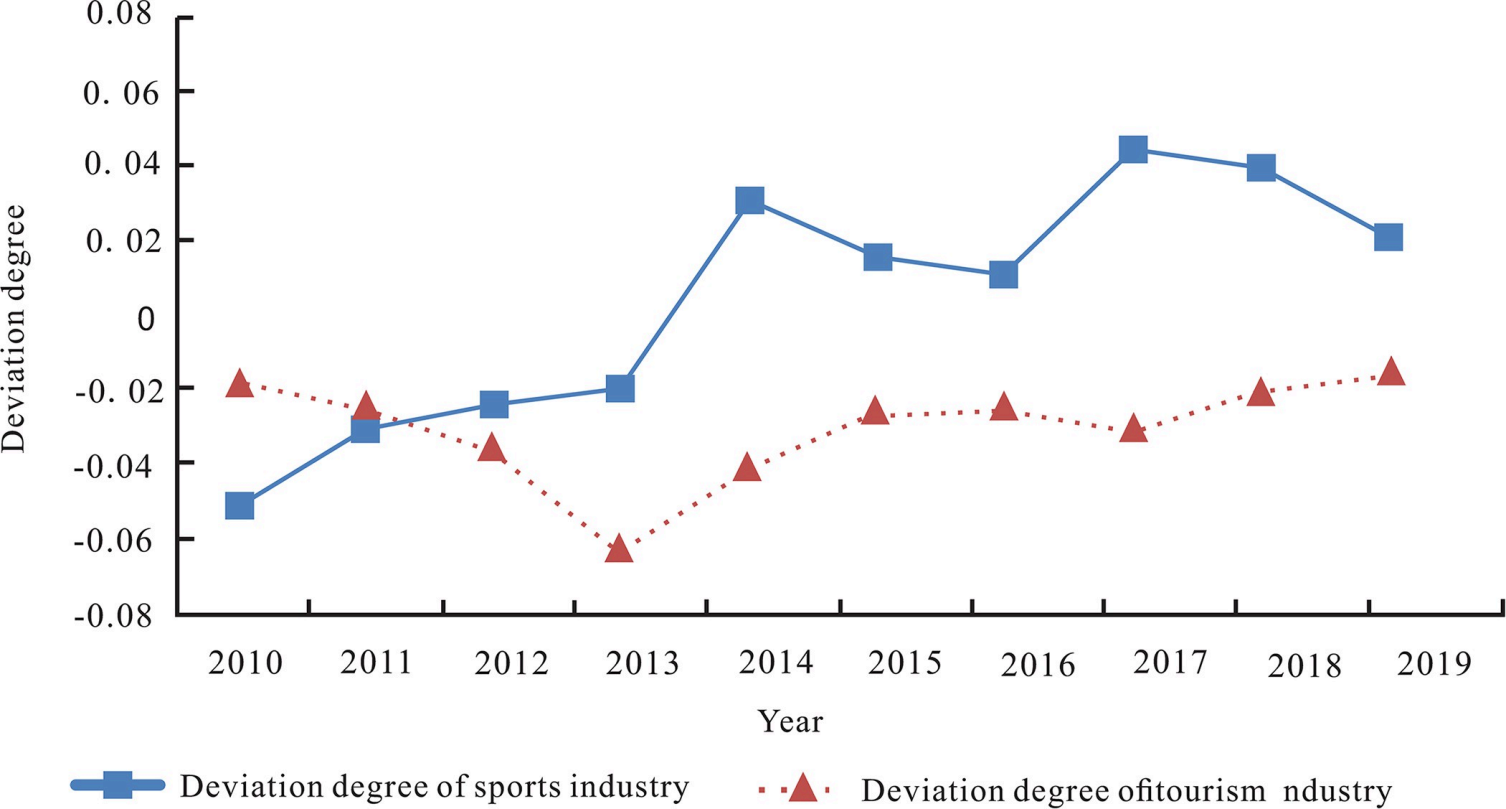
Average deviation degree of sports and tourism industries.

Fig 3 illustrates the trend of the average deviation degree based on Table 4. It shows that the deviation of two industries from their ideal level fluctuate roughly alternately. The actual level of tourism industry from 2010 to 2019 was lower than the ideal value, indicating that the tourism input was relatively small compared with the ideal input. For the sports industry, the actual development level from 2010 to 2013 was lower than the ideal value; while the actual development level of the sports industry was higher than the ideal level from 2014 to 2019, showing that the sports industry input at this stage was larger than the ideal investment. However, taking also Fig 2 into account, we find that the excessive investment in sports industry from 2014 to 2019 did not necessarily significantly improve the integration extent to which sports promotes the development of tourism, which reflects that the excessive investment in one industry does not necessarily improve the level of industrial integration.

#### Spatial characteristics analysis

**Fig 4:**
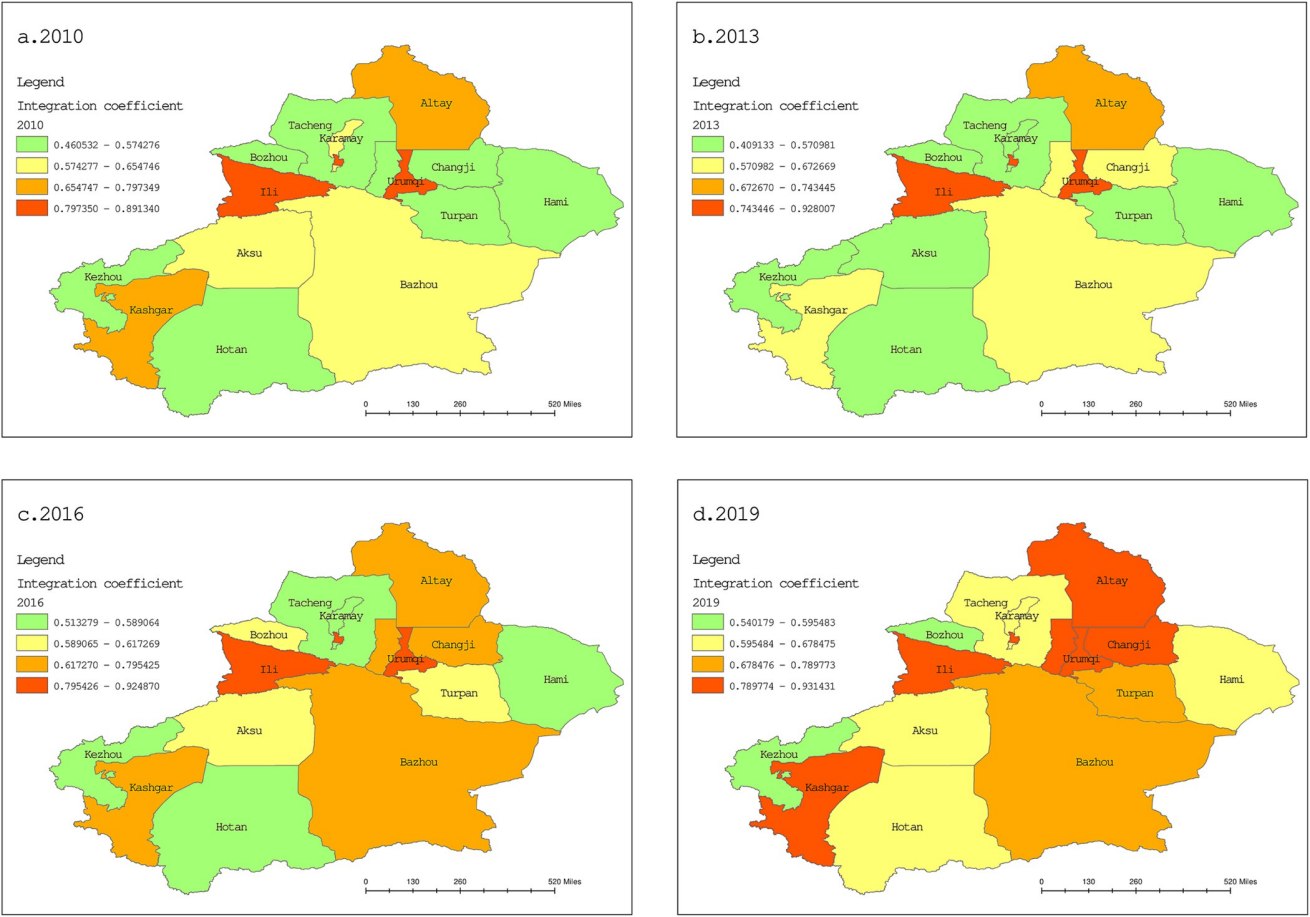
Spatial distribution of industrial integration quality.

In order to more intuitively show the spatial characteristics of industrial integration quality level in various regions, ArcGIS10.8 is used to draw the spatial differentiation graph of the integration quality levels in different regions in 2010, 2013, 2016 and 2019, based on the results in Table 3. The data of DEM is derived from the 1:1,000,000 basic geographic information data, provided by the National Catalogue Service For Geographic Information of China (www.webmap.cn). The diagrams are shown in Fig 4.

From the spatial differences, the integration quality levels presented a phenomenon of “imbalance and inadequacy” in space, and there were big differences in the integration quality among different regions. In 2010, the quality level of industrial integration in Urumqi and Ili Prefecture was relatively higher, followed by Altay and Kashgar; and the quality level of industrial integration in the rest prefectures was relatively lower. In 2013, the industrial integration quality in Changji was improved, while Kashgar and Aksu showed a decline. In 2016, the integration quality level was improved in various prefectures, among which Changji, Turpan, Bozhou, Aksu, Bazhou and Kashgar showed a significant improvement. In 2019, the industrial integration was further strengthened. The integration quality level in Altay, Kashgar and Changji was gradually close to that in Urumqi and Ili Prefectures. The quality level of industrial integration in Hami, Hotan, Karamay and Tacheng was also significantly improved. During the period from 2016 to 2019, the industrial integration quality level in various prefectures was improved rapidly, which may be related to the promulgation of favorable policies at the national level and provincial level as well as the improvement of transportation facilities in various prefectures during this period. In addition, on the whole, the areas with high quality of industrial integration are located in the three major cities of Urumqi, Ili and Kashgar, as well as Altay and Changji, which are rich in sports tourism resources. While the rest of the prefectures are affected by their respective economic development level, tourism resources, sports infrastructure and other factors, resulting in a relatively low-quality level of industrial integration.

### Analysis of regional differences in the integration quality

#### The temporal characteristics of the integration quality of the tourism industrial belt

**Fig 5:**
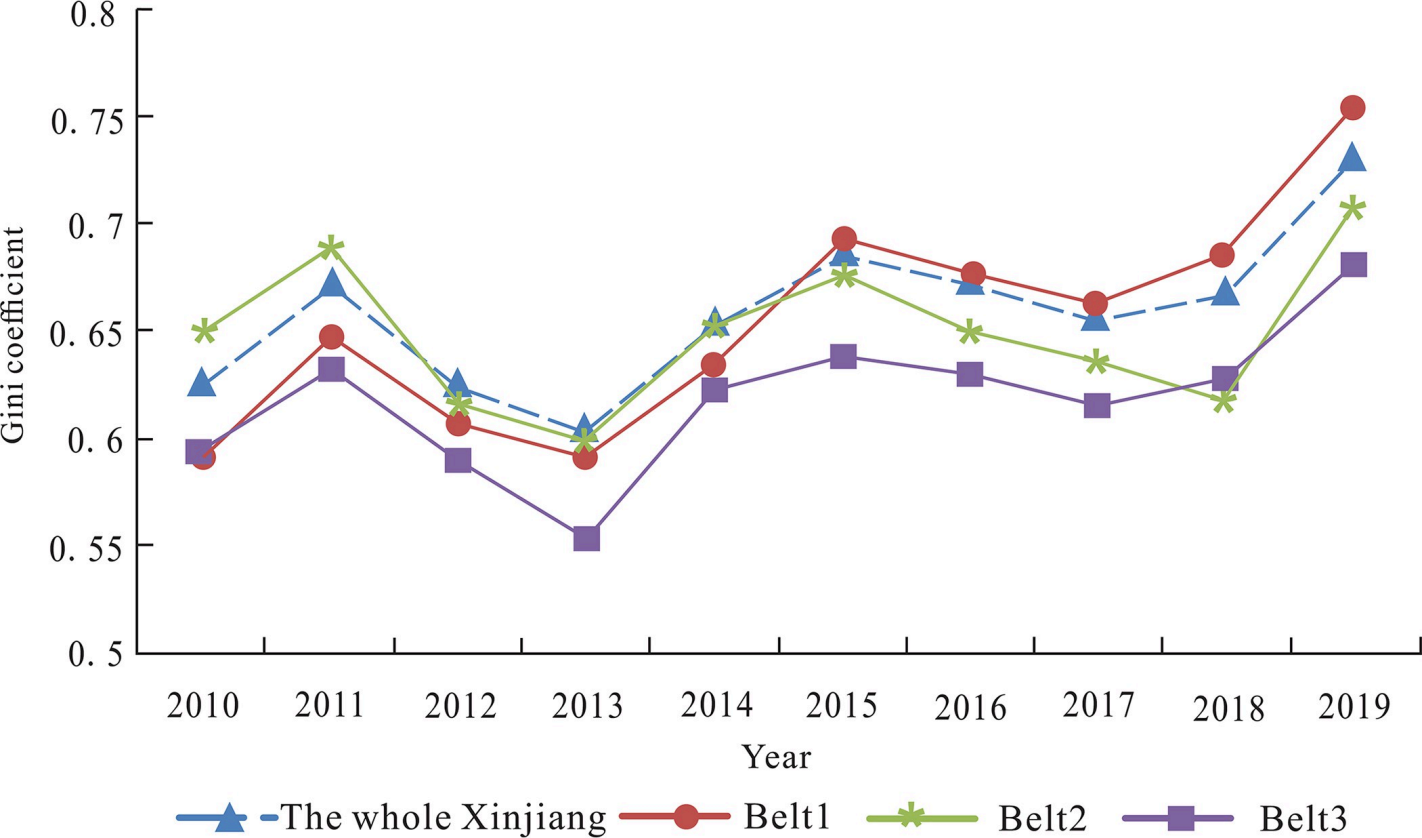
Trend of integration quality level of tourism industrial belts.

According to the “13^th^ Five-year Plan” for Tourism Development in Xinjiang, the 14 regions are grouped into four industrial belts. Industrial Belt 1 is the Tianshan corridor world heritage industrial belt, including Ili, Bozhou, Changji, Turpan, and Hami. Industrial Belt 2 is an Eco-tourism industrial belt in the northern margin of Junggar, including Altay, Tacheng, and Karamay. Industrial Belt 3 is the cultural and ethnic tourism destination along the Silk Road in southern Xinjiang, including Kashgar, Aksu, Bazhou, Hotan, and Kezhou. Industrial Belt 4 is Urumqi International Tourism Distribution Center, including Urumqi alone. Since industrial Belt 4 only includes Urumqi as a tourism distribution center, this paper only examines the other three major tourism industrial belts when analyzing the regional differences of the tourism industrial belt. According to the industrial integration quality of 14 regions in Table 3, we summarize the integration quality level of the corresponding regions, based on the division of industrial belts, and calculate their average value. Therefore, we can get the industrial integration quality level in each tourism industry belt and the average level of Xinjiang (Fig 5).

Seen from Fig 5, the industrial integration quality level in three major tourism industrial belts shows a fluctuating upward trend. Belt 1 displayed the fastest growth in the quality level of industrial integration. Since 2015, it has exceeded the average level of Xinjiang and become the tourism industrial belt with the highest quality level of industrial integration. Due to the slow improvement of the industrial integration quality in Tacheng and Karamay, Belt 2 was slightly lower than the average level of industrial integration quality since 2012, and its gap with the average level of Xinjiang was further widened after 2015. The integration quality level in industrial Belt 3 was the lowest and significantly lower than the average level of Xinjiang. The main reason is that the construction of tourism infrastructure in southern Xinjiang was weak, and the brand building of sports tourism lacked innovation. In addition, the climate and traffic conditions in southern Xinjiang also restricted the development of local sports tourism.

#### Difference analysis of tourism industrial belt

There are some differences in industrial integration quality within and between different tourism industrial belts. We use Dagum Gini coefficient to quantitatively analyze the regional differences of the integration quality level of sports and tourism industries, aiming to achieve balanced development in various tourism industrial belts.

According to the integration quality level in the 14 regions in Table 3, the overall Gini coefficient, intra-regional Gini coefficient and inter-regional Gini coefficient of the integration quality of the whole Xinjiang and the three tourism industrial belts are calculated by Eqs (18), (19) and (21). The measurement results are collected in Table 5.

**Table 5:** Gini coefficient of integration quality in Xinjiang and three tourism industrial belts.

Year	*G*	*G*_*jj*_			*G*_*jh*_		
		Belt 1	Belt 2	Belt 3	Belt 1–2	Belt 1–3	Belt 2–3
2010	0.1152	0.0908	0.0918	0.0967	0.1075	0.1059	0.1061
2011	0.1060	0.0865	0.0815	0.0955	0.0950	0.0978	0.1037
2012	0.1256	0.1142	0.0756	0.1075	0.1100	0.1224	0.1051
2013	0.1325	0.1166	0.0846	0.1053	0.1090	0.1254	0.1138
2014	0.1137	0.0954	0.0813	0.1014	0.0968	0.1119	0.1000
2015	0.0990	0.0762	0.0718	0.0915	0.0808	0.0952	0.0901
2016	0.1036	0.0920	0.0701	0.0916	0.0887	0.0992	0.0883
2017	0.1143	0.1148	0.0762	0.0937	0.1076	0.1127	0.0921
2018	0.1182	0.1132	0.0825	0.0971	0.1156	0.1163	0.0978
2019	0.0964	0.0875	0.0727	0.0878	0.0894	0.1019	0.0860

**Fig 6:**
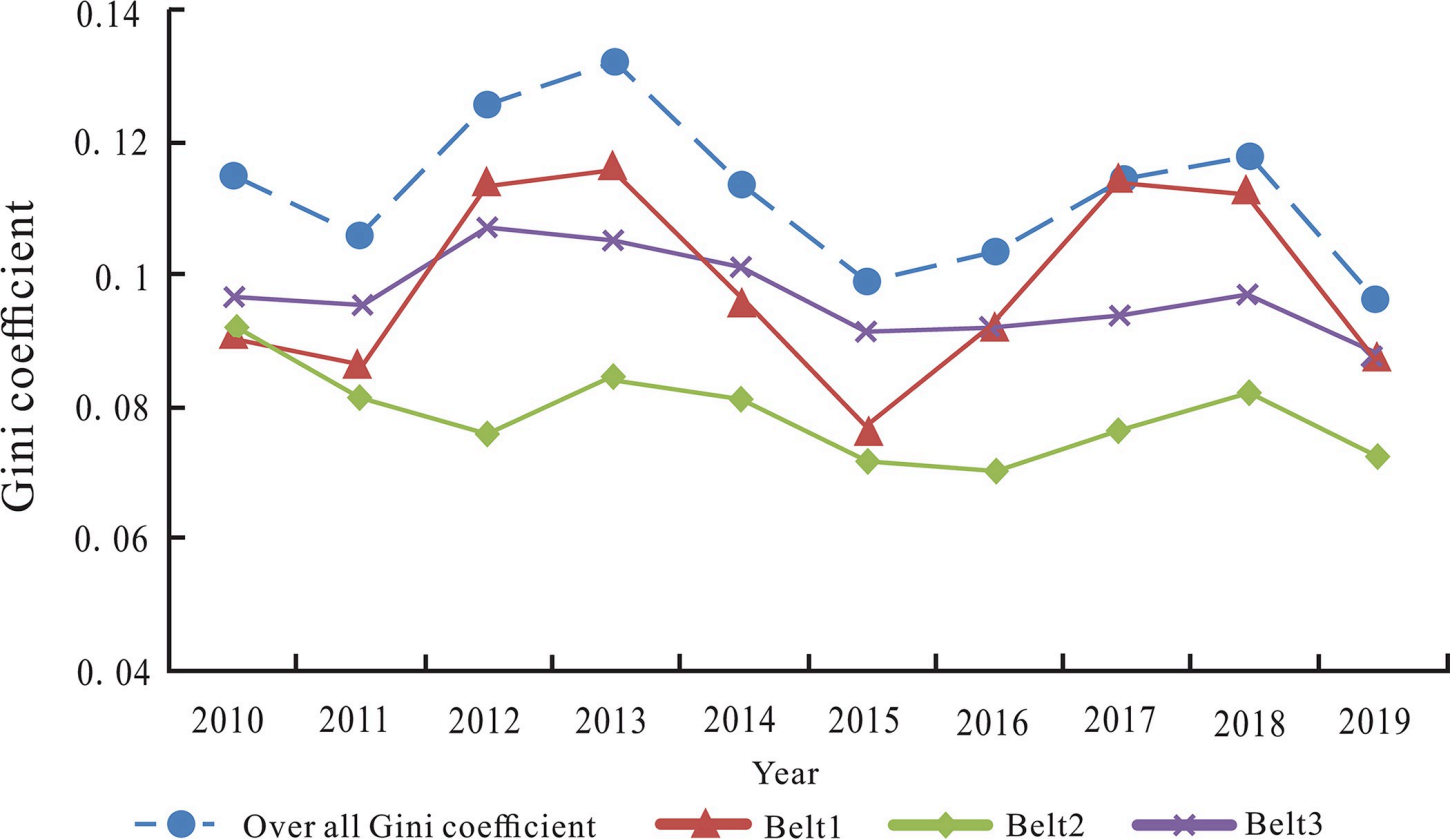
Overall and intra-regional differences of tourism industrial belts.

Based on the results in Table 5 and Fig 6 depicts the overall Gini coefficient of industrial integration quality in three tourism industrial belts and the variation trend of Gini coefficient within the area during the sample period.

As shown in Fig 6, the overall difference of the integration quality in Xinjiang showed a downward trend of fluctuations. From the perspective of internal differences in tourism industrial belts, there were obvious spatial differences in three major tourism industrial belts. Firstly, Belt 2 had the smallest intra-regional differences and showed a certain downward trend. Next, Belt 3 was affected by tourism resources, economic development level, transportation, climate conditions and other factors, resulting in the relatively large regional differences. Finally, the fluctuation degree of intra-regional differences in Belt 1 was the largest. Although the industrial integration quality in Changji was improved rapidly, but was affected by the slow development of Bozhou and Hami, and thus the imbalance between different regions was relatively obvious.

**Fig 7:**
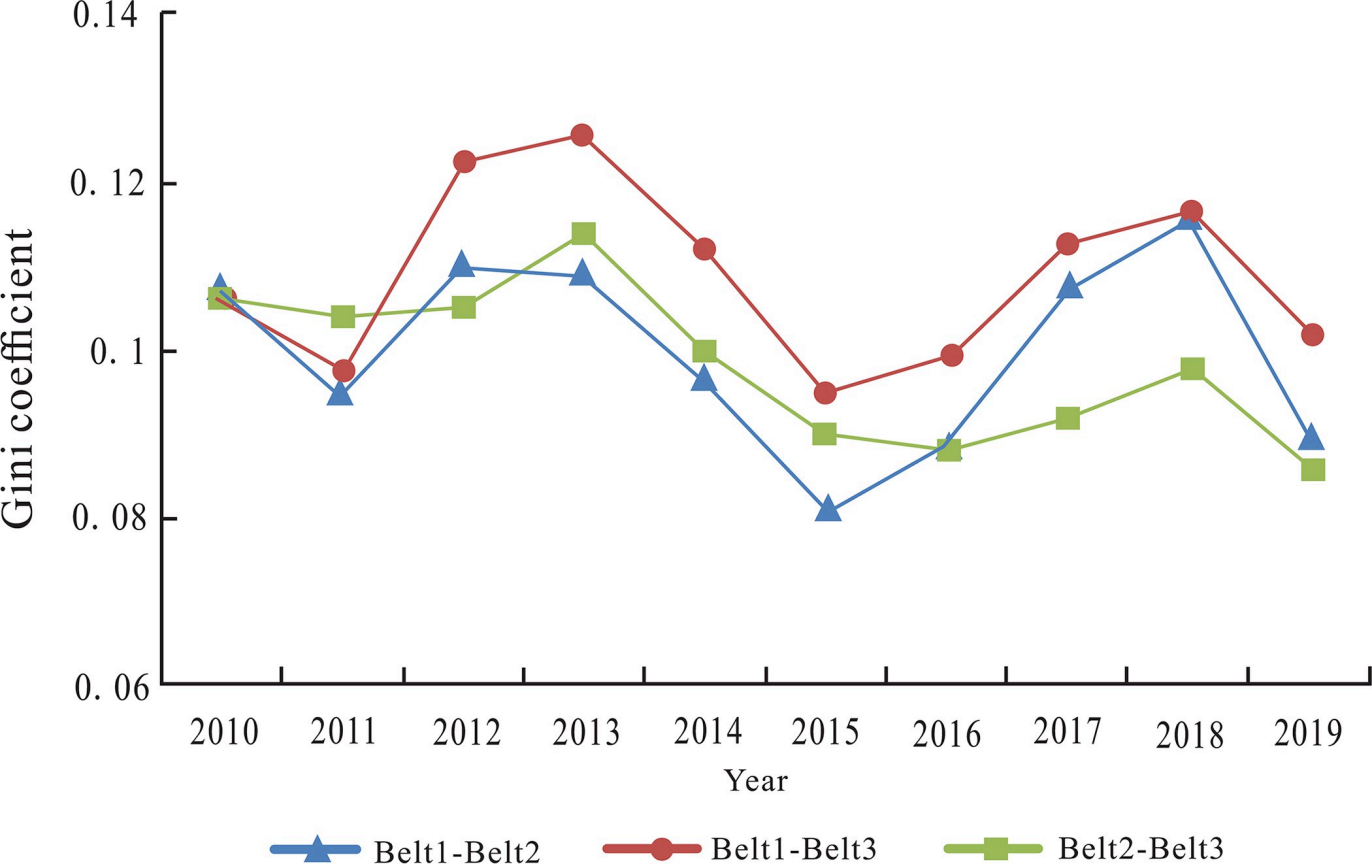
Inter-regional differences of tourism industrial belts.

According to the results in Table 5 and Fig 7 describes the temporal trend of inter-regional Gini coefficient of industrial integration quality among the three tourism industrial belts during the study period. We can see that inter-regional difference between Belt 1 and Belt 3 was the largest. Before 2016, the difference between Belt 1 and Belt 2 was the smallest, showing that the difference of integration quality was affected by the geographical conditions of various prefectures in northern and southern Xinjiang. Due to the arid climate, inconvenient transportation and a lagging-behind tourism infrastructure, the five prefectures in Belt 3 were relatively different from other tourism industrial belts. However, after 2016, the inter-regional difference between Belt 1 and Belt 2 was increased, and it was greater than the difference between Belt 2 and Belt 3. The main reason is that since 2016, due to the rich tourism resources and convenient traffic conditions, the sports tourism industry of the regions within Belt 1 has been developed rapidly benefitted from the policy promotion, which generated a widening gap with the other two major tour-ism industrial belts.

In order to investigate the sources and contributions of the differences in the industrial integration quality level of tourism industrial belts, based on the results in Table 5, the Eqs (20), (22) and (23) are used to calculate intra-regional difference (*G*_*w*_), inter-regional net difference (*G*_*nb*_) and hypervariable density (*G*_t_) of tourism industrial belts, respectively (Table 6).

**Table 6:** The Sources of differences in integration quality.

Year	Intra-Regional Difference		Intra-Regional Difference		Hypervariable Density	
	*G*_*w*_	Contribution-Rate	*G*_*nb*_	Contribution-Rate	*G*_*t*_	Contribution-Rate
2010	0.027	23.45%	0.045	38.93%	0.043	37.62%
2011	0.026	24.54%	0.040	37.44%	0.040	38.02%
2012	0.031	24.28%	0.041	32.81%	0.054	42.90%
2013	0.031	23.225	0.053	39.61%	0.049	37.17%
2014	0.028	24.47%	0.037	32.38%	0.049	43.15%
2015	0.024	24.11%	0.042	42.53%	0.033	33.35%
2016	0.026	24.97%	0.040	38.73%	0.038	36.30%
2017	0.029	25.64%	0.043	37.69%	0.042	36.67%
2018	0.030	25.36%	0.048	40.29%	0.041	34.36%
2019	0.025	26.13%	0.040	41.68%	0.031	32.19%

As shown in Table 6, the main source of spatial differences lies in inter-regional net differences, and the inter-regional net differences had a certain upward trend in recent years. In addition, the contribution of hypervariable density was only slightly lower than the inter-regional net differences, and in some years, it was even greater, indicating the significant influence of hypervariable density on spatial difference.

### Dynamic evolution analysis of the integration quality

This paper uses Dagum Gini coefficient to reveals numerical level and specific sources of regional differences of industrial integration quality in various regions, and identifies the trajectory of relative difference change among various tourism industrial belts, but it cannot describe the evolution process of the integration quality in various regions. Therefore, this paper uses the three-dimensional evolution graph of kernel density to describe the distribution characteristics in each tourism industrial belt.

**Fig 8:**
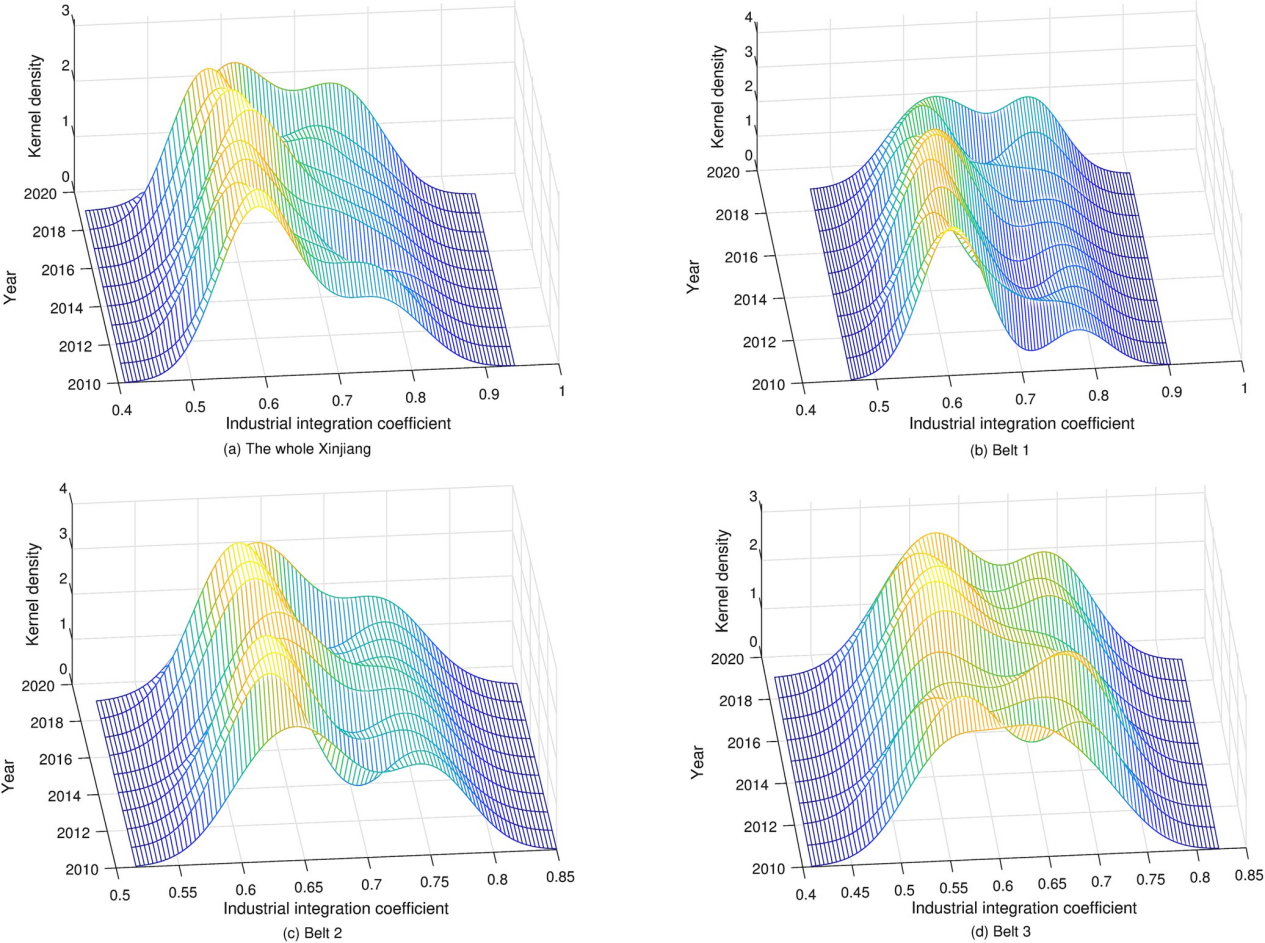
Dynamic evolution of the industrial integration quality.

According to the industrial integration quality level in the 14 regions in Table 3, the kernel density of Xinjiang as a whole and each tourism industrial belt is estimated by Eqs (24) and (25). And draw three-dimensional evolution graph to identify the dynamic evolution process of the industrial integration quality via analyzing the key attributes of the corresponding density curve (Fig 8).

1. From the distribution position, the distribution centers of Xinjiang as a whole and the three tourism industrial belts showed a trend of shifting to the right in general, showing that industrial integration quality in various regions was constantly being improved. However, during the study period, the curve was briefly shifted to the left, which meant that some regions were limited by their own economic development conditions, and there were difficulties in the introduction and implementation of relevant policies, resulting in a relatively lower industrial integration quality level.
2. From the curve peak characteristics, the peak value of industrial integration quality in Xinjiang as a whole was generally on the rise, and the width of the main peak became narrowed, which shows that the gap of industrial integration quality in various regions was decreased. For the three tourism industrial belts, the main peak of the belt 1 showed a phenomenon of fluctuating decline, indicating that the difference of industrial integration quality among different regions in this tourism industrial belt was constantly being widen. The main peak of Belt 2 had relatively little change in height and width. While the main peak of Belt 3 showed a fluctuating rise, which indicates that the difference of industrial integration quality between the five prefectures in Belt 3 became gradually decreased.
3. From the number of peaks, there were double peaks in Xinjiang as a whole and in the three tourism industrial belts during the study period, indicating that there was two-grade distribution in the industrial integration quality level in various prefectures within the region. Among them, the distance between the main peak and secondary peak of Xinjiang as a whole and Belt 1 was decreased, indicating that the two-grade distribution characteristics within the region tended to become weakened in general. While, the two-grade distribution of the industrial Belt 2 was still severe. In industrial Belt 3, the distance between the main peak and the second peak was gradually shorter, showing that the degree of a two-grade distribution between the five prefectures in Belt 3 was gradually weakened.
4. From the distribution ductility, the density curves of Xinjiang as a while, the industrial Belt 1 and Belt 2 all had a significant right trailing phenomenon. It shows that industrial integration quality in some prefectures within the belt was significantly higher than that in other prefectures in the same belt. While, the kernel density curve of Belt 3 had no obvious trailing feature, indicating that the industrial integration quality level of the five prefectures in Belt 3 was more balanced compared with other tourism industrial belts.

## Discussion

Current research on sports tourism integration primarily focuses on theoretical aspects, such as connotation, mechanism, mode, and effects. Some studies also assess the integration level of sports and tourism industries using coupling coordination measures. However, these studies fail to show that the integration of the sports and tourism industries is a dynamic process that involves interaction. Furthermore, there is a lack of in-depth discussion on the integrated development path for sports promoting the tourism industry and tourism driving the sports industry. Moreover, the research area is mostly centered at the national and urban agglomeration levels, overlooking the potential of sports tourism integration at the local and regional levels. In this study, we aim to address this knowledge gap and provide novel insights into the integrated development of sports and tourism industries. Specifically, we have made improvements in the following three aspects.

Firstly, we have evaluated the path for integrated development of sports promoting the tourism industry and tourism driving the sports industry, using the theory of technical efficiency. We found that the two paths are inter-connected, and have defined the quality of sports tourism industry integration as the result of coupling coordination. This dynamic interactive process demonstrates the importance of integrating sports and tourism industries.

Secondly, we chose Xinjiang, the core region of the ’Belt and Road’ initiative, as our research object. With abundant tourism resources, diverse customs, and thriving culture, Xinjiang is undergoing a tourism boom fueled by the strategy of rejuvenation through tourism. Thus, this study explores the regional-level integrated development of sports tourism in Xinjiang.

Finally, we have considered the tourism development layout outlined in ’the 13th Five-Year Plan’ of Xinjiang’s tourism industry. The plan divides Xinjiang’s 14 prefectures and cities into four industrial belts, each with its own unique tourism focus. By aligning our research with this regional strategy, we aim to provide insights into the integrated development of sports tourism that can contribute to the sustainable growth of the tourism industry in Xinjiang and beyond.

By adopting an industrial belt perspective, this paper provides a detailed analysis of regional differences in the quality of integrated sports tourism industry. Our findings offer valuable insights to policymakers and tourism industry managers on how to strengthen regional cooperation and effectively manage tourism industrial belts. By conducting a quantitative analysis of the integration quality of the sports tourism industry, this study presents reliable and effective research findings. These results offer a scientific basis for devising regional development plans for Xinjiang’s sports tourism industry and address the subjectivity and one-sidedness limitations of previous qualitative research. Currently, sports tourism development in Xinjiang is in its initial stage, with tourism economic income predominantly reliant on scenic spots and local tourism resources. However, organic integration with ethnic minority sports and cultural resources has yet to be fully achieved.

Based on the results of the study, several management implications are proposed:

1. The local government or authority should pay attention to the construction of the sports infrastructures. It should be encouraged for the prefectures to strive for the right to host various sports events. Furthermore, we should combine sport activity with local scenic spots and culture to create folk sports events with regional characteristics. For the existing event resources, each prefecture or city should increase investment on the events and raise their publicity to attract more participants and tourists. With the help of new media resources such as short videos and public accounts, each prefecture or city should create influential sport events. At the same time, each prefecture or city should fully implement the policy of opening stadiums for free or with low renting fees to realize the goal of national fitness campaign more quickly.
2. The local government or authority should take the leadership to create the distinctive sports tourism brands. In the regions where the sports tourism industry is being developed slowly, we should strengthen the policy and financial support, speed up the development of sports tourism products based on the natural and cultural resources of regional characteristics. Within each tourism industrial belt, we should take advantage of the prefectures with high quality of industrial integration, strengthen the communications and cooperation among the prefectures, and realize the effective allocation of regional sports.
3. The government or authority should plan the travel route reasonably to provide more convenient and quicker transportation to promote balanced development. The government or authority should increase transportation or travelling facilities with higher standards to promote the coordinated development of regional sports tourism.

## Conclusions

This study measures the industrial integration quality in the 14 regions of Xinjiang from 2010 to 2019 through entropy method, stochastic frontier analysis, and coupled coordination model. Furthermore, the Dagum Gini coefficient and Kernel density estimation are used to empirically analyze the regional differences of industrial integration quality of each tourism industry belt. The major conclusions are as follows:

1. From 2010 to 2019, the industrial integration quality in Xinjiang did not reach the optimal goal of complete integration. For the two integration paths, the integration degree of tourism driving sports industry was higher than the integration degree of sports promoting tourism.
2. From the deviation from the frontier, the path deviation extent of sports promoting tourism is greater than that of tourism driving sports. In terms of deviations from the ideal level, the deviation of sports and tourism from their ideal level showed alternating fluctuation.
3. The industrial integration quality in Xinjiang showed a phenomenon of “imbalance and inadequacy” among the regions. The regions with high quality of industrial integration included Urumqi, Ili, Kashgar, Altay and Changji, which are the places with rich sports tourism resources.
4. The overall difference of industrial integration quality in Xinjiang showed a downward trend of fluctuations. From the tourism industrial belts, firstly, the intra-regional difference of Eco-tourism industrial belt in the northern margin of Junggar was the smallest, followed by five prefectures in southern Xinjiang. In addition, the inter-regional difference between the Tianshan corridor world heritage industrial belt and the five prefectures in southern Xinjiang was the largest. Finally, the main source of spatial differences lies in inter-regional net difference.
5. There was an obvious two-grade differentiation about the industrial integration quality level in various prefectures and in three tourism industrial belts. In addition, in the Tianshan corridor world heritage industrial belt and the Eco-tourism industrial belt in the northern margin of Junggar, the industrial integration quality in some regions within tourism industrial belt was significantly higher than that in other regions in the same industrial belt. While, the industrial integration quality level of the five prefectures in southern Xinjiang was more balanced compared with other tourism industrial belts.

Although this study provides a new framework for the evaluation of the integrated development of sports tourism in Xinjiang, there are still some limitations that need to be addressed. For example, due to limitations in the availability of indicator data, the representativeness of the research project has decreased. Secondly, the impact of "spatial lag" has not been taken into account. In the future, it is possible to optimize the indicator system and explore the factors influencing the unbalanced development of the integrated sports tourism industry from the perspective of spatial spillover effect. Furthermore, future research can be done to investigate the industrial structure and regional distribution of integrated traditional ethnic sports and tourism, as well as to explore the potential of sports tourism to spur regional development. This will enable more effective development of traditional ethnic sports products and the creation of unique sports tourism intellectual property in Xinjiang. Finally, we can investigate how the organic fusion of sports and tourism can promote related industries, particularly in achieving the objective of sports tourism driving multi-industry development.
